# Genome sequencing of four culinary herbs reveals terpenoid genes underlying chemodiversity in the Nepetoideae

**DOI:** 10.1093/dnares/dsaa016

**Published:** 2020-07-31

**Authors:** Nolan Bornowski, John P Hamilton, Pan Liao, Joshua C Wood, Natalia Dudareva, C Robin Buell

**Affiliations:** 1 Department of Plant Biology, Michigan State University, East Lansing, MI 48824, USA; 2 Department of Biochemistry, Purdue University, West Lafayette, IN 47907-2063, USA; 3 Plant Resilience Institute, Michigan State University, East Lansing, MI 48824, USA; 4 MSU AgBioResearch, Michigan State University, East Lansing, MI 48824, USA

**Keywords:** Lamiaceae, Nepetoideae, genome assembly, terpenoid synthase, comparative genomics

## Abstract

Species within the mint family, Lamiaceae, are widely used for their culinary, cultural, and medicinal properties due to production of a wide variety of specialized metabolites, especially terpenoids. To further our understanding of genome diversity in the Lamiaceae and to provide a resource for mining biochemical pathways, we generated high-quality genome assemblies of four economically important culinary herbs, namely, sweet basil (*Ocimum basilicum* L.), sweet marjoram (*Origanum majorana* L.), oregano (*Origanum vulgare* L.), and rosemary (*Rosmarinus officinalis* L.), and characterized their terpenoid diversity through metabolite profiling and genomic analyses. A total 25 monoterpenes and 11 sesquiterpenes were identified in leaf tissue from the 4 species. Genes encoding enzymes responsible for the biosynthesis of precursors for mono- and sesqui-terpene synthases were identified in all four species. Across all 4 species, a total of 235 terpene synthases were identified, ranging from 27 in *O. majorana* to 137 in the tetraploid *O. basilicum*. This study provides valuable resources for further investigation of the genetic basis of chemodiversity in these important culinary herbs.

## 1. Introduction

The Lamiaceae (mint) family is among the largest angiosperm families, containing ∼7,000 species that occupy a wide geographic distribution^1^ and are commonly recognized by their square stems, opposite leaves, and lobed inflorescences. The Lamiaceae is not only rich in species number and diversity, but also in the production of specialized metabolites. The largest class of these metabolites, terpenes, exhibit substantial chemodiversity and broad variation in abundances across Lamiaceae.^2^^,^^3^ Recent molecular-based phylogenetic analyses support 10 − 12 major clades within the mint family, the largest of which is the Nepetoideae that contains approximately half of all Lamiaceae species.^3^^,^^4^ The Nepetoideae also has the greatest diversity of monoterpenes^5^ among the mint clades, making it a robust clade to study the relationship between terpenoid diversity and species diversity.

All terpenoids are derived from two universal precursors, isopentenyl diphosphate (IPP) and its isomer dimethylallyl diphosphate (DMAPP), which are synthesized in plants via two independent pathways: the methylerythriol phosphate (MEP) pathway in the plastid and the mevalonic acid (MVA) pathway distributed among the cytosol endoplasmic reticulum and peroxisomes. IPP and DMAPP then serve as substrates for short-chain prenyltransferases, which produce the prenyl diphosphates, geranyl diphosphate (GPP) in the plastid and farnesyl diphosphate (FPP) in the cytosol. Finally, GPP and FPP are converted into monoterpenes and sesquiterpenes, respectively, by the action of enzymes of the terpene synthase (TPS) superfamily. Product promiscuity of TPSs and enzymes modifying TPS products are the main sources of terpene structural diversity in plants.^6^

A number of Nepetoideae species are used as culinary herbs due to their production of specialized metabolites that impart unique flavour profiles. Sweet basil (*Ocimum basilicum* L.), e.g. exhibits a wide range of phenotypes and chemotypes^7^ and is commonly used in pesto sauce. Although ploidy varies within *O. basilicum*, the cultivar ‘Genovese’ is tetraploid (2*n* = 4*x* = 48) with a genome size of 4.1 − 4.7 Gb estimated by flow cytometry.^8^^,^^9^ Rosemary (*Rosmarinus officinalis* L.), in contrast, is an evergreen shrub with grey−green needle-like leaves that emit a strong fragrance due to multiple aromatic volatiles.^10^^,^^11^*Rosmarinus officinalis* and its essential oils have been widely used in cultural practices, culinary flavourings, medicinal remedies, and pest deterrents.^12^ Oregano (*Origanum vulgare* L.) and sweet marjoram (*Origanum majorana* L.) are both members of the *Origanum* genus, having a shrub-like architecture and small, ovate leaves. Their relatedness is evidenced biologically and culturally as *Origanum* spp. can make interspecific hybridizations^13^ and some regions of the world refer to oregano and marjoram interchangeably. Young leaves of both *Origanum* spp. are harvested, dried, ground, and added to culinary dishes to bestow a spicy, bitter-sweet flavour. Three major chemotypes have been described for *O. vulgare*: acyclic, cymyl, and sabinyl.^14^ Chemotypes of *O. majorana* are less-defined due to nomenclature challenges and the presence of distillation artefacts^15^^,^^16^ but both cymyl and sabinyl compounds have been widely documented.^17^^,^^18^

All four species were part of the 1k Plant Transcriptome Initiative^19^^,^^20^ and leaf transcriptomes were generated to examine the evolution of green plants as well as for understanding the evolution of chemodiversity within the Lamiaceae.^3^ In addition, for *O. basilicum*, transcriptomes were generated for cultivars ‘Tiguillo’ and ‘Red Rubin’^21^ as well as ‘CIM Saumya’,^22^ and a genome assembly of the *O. basilicum* cultivar ‘Perrie’ has recently been reported although the actual sequence is not currently available.^23^ There is a growing number of Lamiaceae species with assembled genomes (see Supplementary Dataset S1) that have enabled identification of genes involved in specialized metabolism and a broader understanding of genome organization with respect to specialized metabolism.^24^ However, the genetic repertoire encoding chemical diversity within the culinary herbs remain largely unexplored. Here, we here report the genome sequence, annotation, and metabolite profiling of four culinary herbs and describe their repertoire of terpenoid biosynthetic genes that will provide a resource for data-mining not only terpenoid biosynthetic pathway genes but also other genes that function in specialized metabolism.

## 2. Materials and methods

### 2.1. Plant materials and growing conditions

Plant samples for *O. basilicum* ‘Genovese’ and *R. officinalis* ‘Arp’ were purchased from VanAtta’s Greenhouse and Flower Shop (Haslett, MI), whereas *O. majorana* and *O. vulgare* were obtained from Richter’s Herbs (Canada). *Ocimum basilicum* and *R. officinalis* were grown in a growth chamber under a 14-/10-h day/night cycle with a daytime temperature of 27°C and a night-time temperature of 15°C; light intensity in the chamber was 210 μE m^−2^ s^−1^. *Origanum majorana* and *O. vulgare* were grown in a greenhouse under a 15-/9-h day/night cycle at a temperature of 26.6°C. Plant management included weekly fertilizing and pesticide application as necessary. Flow cytometry was performed on leaf samples at the Benaroya Research Institute (Seattle, WA), and *k*-mer estimated genome sizes were determined using Jellyfish v2.2.0^25^ with a *k*-mer size of 31 and adjusted for heterozygous sequences using the R package findGSE v0.1.0.^26^

### 2.2. DNA and RNA isolation

Nuclei were isolated from leaf tissue following a previously described protocol^27^ with an input of 1 − 2 g of ground tissue; spin speeds were used based on estimated genome size, 2,700, 3,030, 3,030, and 2,900 g for *O. basilicum*, *O. majorana*, *O. vulgare*, and *R. officinalis*, respectively. DNA was extracted using the Nanobind Plant Nuclei Big DNA Kit (Circulomics, Baltimore, MD, Cat No. NB-900-801-01). RNA was extracted from mature leaf tissue using a hot phenol protocol,^28^ and DNA was removed using the TURBO DNA-*free™* Kit (Invitrogen, Carlsbad, CA, Cat No. AM1907). Quality and concentrations were verified by Nanodrop, Qubit, and agarose gel electrophoresis.

### 2.3. Library construction, sequencing, and expression abundance estimation

Genomic libraries were constructed using 10× Genomics Technology (Chromium™ Genome Library Kit & Gel Bead Kit v2; Pleasanton, CA, USA) and sequenced at the Roy J. Carver Biotechnology Center at the University of Illinois at Urbana-Champaign. Sequencing was performed on an Illumina NovaSeq 6000 at 150 nt in paired-end mode. Libraries were pooled with an aim of 65× coverage for each species. RNA-Seq libraries were constructed using the Illumina TruSeq Stranded mRNA Kit with polyA mRNA selection and IDT for Illumina Unique Dual Index primers (Illumina, San Diego, CA, USA) and sequenced at the Michigan State University Research Technology Support Facility. RNA-Seq libraries were sequenced on an Illumina HiSeq 4,000 at 150 nt in paired-end mode. Reads were cleaned with Cutadapt v2.3,^29^ which trimmed adapters and 3′ bases with a quality score <10, and only kept reads of at least 100 nt. After cleaning, reads were aligned to their respective genomes using HISAT2 v2.1.0^30^ with the following parameters set: –dta-cufflinks, –max-intronlen 5000, and –rna-strandness RF. Cufflinks v2.2.1^31^ was run in stranded mode to generate expression abundances (fragments per kilobase of exon model per million mapped reads).

### 2.4. Genome assembly and annotation

The 10× Genomics reads were demultiplexed and assembled using Supernova v2.1.1,^32^ with –maxreads set to 900 million, 330 million, 259 million, and 450 million reads for *O. basilicum*, *O. majorana*, *O. vulgare*, and *R. officinalis*, respectively. Scaffolds containing only N sequences were removed from the final assemblies. Custom repeat libraries (CRL) were generated for each species using RepeatModeler v1.0.8 (http://www.repeatmasker.org; last accessed April 2020) as described previously.^33^ Genome assemblies were masked with their respective CRLs using RepeatMasker v4.0.6 (http://www.repeatmasker.org; last accessed April 2020). Gene prediction on the masked assembly was performed using Augustus v3.1^34^ with a matrix trained for the Nepetoideae species, *Hyssopus officinalis* L.^35^ To refine the gene models, leaf RNA-Seq libraries were cleaned and used to generated genome-guided transcript assemblies using Trinity v2.3.2^36^ with a maximum intron size of 5,000 and a minimum contig length of 500 nt in stranded mode. The genome-guided transcript assemblies were used with PASA2 v2.3.3^37^ to create the working gene model set. To identify high confidence gene models from the working gene model set, the gene models were searched against PFAM v32.0^38^ using HMMER v3.2.1 (hmmer.org) with search cut-offs–domE 1e−3 −E 1e−5, and gene abundances of the leaf RNA-Seq library were calculated using Kallisto v0.46.0.^39^ Gene models that were not partial models, did not contain an internal stop codon, not transposable element-related, and had a PFAM domain match or a TPM > 0 were selected as high confidence models. Functional annotation of the high confidence gene models was generated as described previously.^33^ Gene ontology (GO) terms were assigned to the representative high confidence gene models using IPRscan v5.34.73.0.^40^

### 2.5. Genome sequence and annotation quality assessment

To assess the completeness of the assembly, whole genome shotgun libraries were processed using Cutadapt v2.3^29^ and reads were aligned to their respective assemblies with BWA-MEM v0.7.16a.^41^Paired-end RNA-Seq libraries constructed from leaf tissue were aligned to the assemblies using HISAT2 v2.1.0^30^ using stranded mode with a maximum intron length of 5,000 bp. Coverage of the genic space was assessed using BUSCO v3.0.2b^42^^,^^43^ with the Embryophyta odb9 dataset (creation date: 2016-02-13, number of species: 30, number of BUSCOs: 1,440) to detect conserved orthologs in the assemblies.

### 2.6. Extraction and analysis of terpenoids by gas chromatography−mass spectrometry

The same leaf tissue harvested for RNA extraction was used for terpenoid profiling. Tissue from each species (0.2 g) was ground in liquid nitrogen and extracted overnight with shaking at room temperature with 5 ml of dichloromethane containing 6.6 μg of the internal standard naphthalene. After centrifugation, the solvent containing the extracted metabolites was transferred to a new glass tube and concentrated to ∼180 µl under nitrogen gas.^44^ Subsequently, gas chromatography−mass spectrometry (GC−MS) analysis was performed on an Agilent 6890 gas chromatograph (Agilent Technologies) equipped with a HP-5MS column (30 m, 0.25 mm, 0.25 μm; Agilent Technologies) and coupled to an Agilent 5975B insert MSD quadrupole mass spectrometer (Agilent Technologies). Each sample (2 μl) was injected at a pulsed splitless mode at 250 °C. The column temperature was held at 50 °C for 2 min, followed by increased to 320 °C at 20 °C min^−1^, and held at 320 °C for 4.5 min. Helium was applied as a carrier gas at a flow rate of 1 ml min^−1^. MS ionization energy was set at 70 eV, and the mass spectrum was scanned from 50 to 300 amu. Three biological replicates were used for metabolite profile analysis for each species. Compounds were identified by comparing retention times and mass spectra with those of commercially available authentic standards including α-pinene, β-pinene, α-phellandrene, α-terpinene, *cis*-β-ocimene, γ-terpinene, terpinolene, linalool, geraniol, β-caryophyllene, and caryophyllene oxide as well as by comparing mass spectra to the National Institute of Standards and Technology (NIST) Mass Spectral Library v2.2. Quantification of terpenoids was performed using the Mass Hunter quantitative software (Agilent Technologies, v. B. 07.01) using response factors relative to the internal standard determined experimentally for the commercially available authentic standards α-pinene (representative monoterpene for α-thujene, α-pinene, camphene), β-pinene (representative monoterpene for β-pinene and β-myrcene), α-phellandrene (representative monoterpene for α-phellandrene and β-phellandrene), α-terpinene (representative monoterpene for α-terpinene, γ-terpinene, o-cymene, *cis*-β-ocimene, terpinolene), geraniol (representative monoterpene alcohol), β-caryophyllene (representative sesquiterpene), and nerolidol (representative sesquiterpene alcohol) and normalized to the fresh weight of the tissue.

### 2.7. Comparative genome analyses

Representative peptides from teak (*Tectona grandis* L.f.)^33^ and *Arabidopsis thaliana* Col-0^45^ were included in comparative genome analysis as outgroups for Nepetoideae and Lamiaceae, respectively. Predicted teak peptides were downloaded from GigaDB (http://dx.doi.org/10.5524/100550) on 26 November 2019 and *A. thaliana* peptides were downloaded from Araport11 on 13 November 2019. Orthofinder2 v2.3.7^46^ was run using default settings to identify orthologous and paralogous TPSs in each species. Orthologous groups represented by all species were used to construct and root a consensus species tree with the STAG^47^ and STRIDE^48^ algorithms, respectively. Gene family expansion and contraction were determined with CAFE v4.2.1^49^ using default settings and an ultrametric tree rooted at 125 million years ago based on estimates from multiple studies.^50–53^ Enrichment of GO terms was performed using the Bioconductor package TopGO v2.38.1.^54^

### 2.8. Identification of TPS orthologs

Manually reviewed cloned TPS genes from Lamiaceae were retrieved from SwissProt (Supplementary Table S1). TPSs were selected to include species within and outside the Nepetoideae subfamily of interest, as well as discrete clades within the Nepetoideae. Lamiaceae TPSs were used along with annotated *A. thaliana* TPSs to identify putative orthologs in the predicted proteomes of the four culinary herbs.

### 2.9. Data availability

Raw sequences are available in the National Center for Biotechnology Information Sequence Read Archive under BioProject PRJNA592145. Large files associated with the genomes including genome sequence, annotation, gff, and expression matrices are available in the Dryad Digital Repository under doi https://doi.org/10.5061/dryad.jwstqjq6t.

## 3. Results and discussion

### 3.1. Genome assembly and annotation

Flow cytometry of the four culinary herbs revealed estimated haploid genome sizes consistent with previous studies. The flow cytometry haploid genome estimation of the tetraploid *O. basilicum* var. ‘Genovese’ was 2.34 Gb which is within previous estimates of 2.04 Gb^8^ to 2.37 Gb.^9^ The flow cytometry estimate of haploid genome size for *O. majorana* (880.2 Mb) is comparable to a recent estimate of 846 Mb,^55^ and estimates for *O. vulgare* (694.38 Mb) and *R. officinalis* (1198.05 Mb) are remarkably similar to previously published flow cytometrical estimates of 684.6 Mb^56^ and 1198.05 Mb,^57^ respectively. The *k*-mer estimated genome sizes for *O. basilicum*, *O. majorana*, *O. vulgare*, and *R. officinalis* were 2.15 Gb, 760.95, 665.08, and 1013.85 Mb, respectively (Supplementary Table S2 and Fig. S1); overall, estimation of genome sizes between flow cytometry and *k*-mer frequency were comparable. We utilized Supernova^32^ to assemble the genomes of the four species. As shown in Table 1, reconstituted molecule lengths ranged from 36.91 kb (*O. vulgare*) to 83.47 kb (*R. officinalis*). Assembled contig N50 lengths ranged from 21.82 kb (*R. officinalis*) to 48.30 kb (*O. basilicum*), while scaffold N50s ranged from 368.74 kb (*R. officinalis*) to 1.51 Mb (*O. basilicum*). The GC content of the assemblies varied from 38.12% (*R. officinalis*) to 40.32% (*O. vulgare*). Detection of heterozygous SNPs by the Supernova assembly process ranged from 192 to 1,490 bp in *R. officinalis* and *O. basilicum*, respectively. For the final genome assembly, scaffolds <10 kb were removed resulting in final assembly sizes of *O. basilicum* (2.07 Gb), *R. officinalis* (1.01 Gb), *O. majorana* (760.89 Mb), and *O. vulgare* (630.04 Mb).

**Table 1:** Assembly metrics of four culinary herbs

	*Ocimum basilicum*	*Origanum majorana*	*Origanum vulgare*	*Rosmarinus officinalis*
Ploidy	2*n* = 4*x* = 48^a^	2*n* = 2*x* = 30^b^	2*n* = 2*x* = 28, 30, 32^b^	2*n* = 20, 24^b^
Estimated haploid genome size (flow cytometry) (Mb)	2337.42	880.20	694.38	1198.05
Estimated genome size (Supernova) (Mb)	2360	858.29	705.35	1180
Assembled genome size (Supernova) (Mb)	2067.62	760.89	630.04	1013.85
Mean molecule length (kb)	51.26	43.28	36.91	83.47
Mean distance between heterozygous SNPs (bp)	1490	1340	273	192
Number of scaffolds ≥10 kb	17105	8763	13832	23035
N50 contig size (kb)	48.30	35.95	26.28	21.82
N50 scaffold size (kb)	1506.96	1383.40	157.94	368.74
Assembly GC content	38.42%	40.17%	40.32%	38.12%
Assembly N content	14.65%	11.70%	8.50%	18.67%

a Carović-Stanko *et al*.^8^

b Rice *et al.*^58^

Although the Supernova assembler was originally designed for human genomics applications, it has been used to assemble non-human animal species like perch^59^ and rice coral,^60^ as well as diploid plant species such as pepper,^61^ snowberry,^62^ and maize.^63^ Supernova assemblies have been generated for polyploid species including proso millet (*Panicum miliaceum*), an allotetraploid,^63^ and potato (*Solanum tuberosum* subsp. *andigena*), an autopolyploid.^64^ Our successful assembly of *O. basilicum* further supports the use of Supernova to generate quality assemblies of polyploid species.

To assess the completeness and representation of genic sequences, whole genome shotgun and RNAseq reads were aligned to their cognate genome assembly. At least 95.8% of whole genome shotgun reads aligned to the assemblies (Supplementary Table S3); properly paired reads with correct orientation ranged from 79.0% (*R. officinalis*) to 85.1% (*O. basilicum*). Leaf RNA-Seq reads had overall alignment rates ranging from 82.7% (*O. basilicum*) to 89.4% (*O. majorana*) (Supplementary Table S4). BUSCO analysis of the *O. majorana*, *O. vulgare*, and *R. officinalis* assemblies revealed 89.5 − 90.1% complete orthologs while the *O. basilicum* assembly contained 86.7% of complete orthologs (Table 2). Approximately half of the *O. basilicum* orthologs were present as multiple copies, consistent with its tetraploidy.^8^

**Table 2:** Representation of genic space in four culinary herb genome assemblies as revealed through Benchmarking Single Copy Orthologs (BUSCO)^a^

Species	Complete	Single copy	Duplicated	Fragmented	Missing	Total
*Ocimum basilicum*	1248 (86.7%)	439 (30.5%)	809 (56.2%)	39 (2.7%)	153 (10.6%)	1440
*Origanum majorana*	1289 (89.5%)	877 (60.9%)	412 (28.6%)	30 (2.1%)	121 (8.4%)	1440
*Origanum vulgare*	1297 (90.1%)	1218 (84.6%)	79 (5.5%)	38 (2.6%)	105 (7.3%)	1440
*Rosmarinus officinalis*	1297 (90.1%)	1216 (84.4%)	81 (5.6%)	38 (2.6%)	105 (7.3%)	1440

a Simão *et al*.^43^; Waterhouse *et al.*^42^

Of the species assembled in this study, a previous assembly was reported for *O. basilicum* cultivar, ‘Perrie’^23^ while the present study assembled the cultivar ‘Genovese’. Both cultivars are tetraploid (2*n* = 4*x* = 48) yet flow cytometry estimates of haploid genome size differ; ‘Perrie’ was estimated at 1.59 Gb,^59^ while our flow cytometry estimate of ‘Genovese’ was 2.34 Gb is in agreement with previous estimations^8^^,^^9^ and estimated size using *k*-mer frequency as well as from the Supernova programme. The ‘Perrie’ assembly was 2.13 Gb and the ‘Genovese’ assembly size generated in this study was 2.07 Gb; these differences may reflect variation in genome size among and within *Ocimum* spp.^8^ as well as the level of heterozygosity in the sequenced genomes. Dudai *et al.*^23^ report ‘Perrie’ as highly homozygous while our ‘Genovese’ sample was heterozygous. The ‘Genovese’ N50 contig length was slightly larger than the Dudai *et al.* ‘Perrie’ assembly (48.30 − 45.71 kb, respectively), although the N50 scaffold size was substantially smaller (1.51 − 19.30 Mb) (Supplementary Table S5). Assessment of genic completeness using 1,440 BUSCO genes revealed 93.0 and 86.7% of complete genes in ‘Perrie’ and ‘Genovese’, respectively; however, our ‘Genovese’ assembly contained 30.5% of these genes as single-copy compared with 18.5% for the ‘Perrie’ assembly.

The four genomes were annotated using the gene finder Augustus^34^ and the resulting gene models were refined with PASA2^37^ using the genome-guided transcript assemblies. The initial working gene model sets were filtered for high confidence genes using expression evidence and/or PFAM domains, resulting in high confidence gene models for *O. basilicum* (*n* = 78,990), *O. majorana* (*n* = 33,929), *O. vulgare* (*n* = 32,623), and *R. officinalis* (*n* = 51,389) (Supplementary Table S6). The annotation data sets were assessed for completeness using BUSCO, revealing a high proportion of complete single copy orthologs. Specifically, single copy orthologs were considered complete in the high confidence representative gene model set at frequencies of 88.3% (*O. basilicum*), 90.6% (*O. majorana*), 89.7% (*O. vulgare*), and 89.2% (*R. officinalis*). As expected, the tetraploid *O. basilicum* gene model sets contained substantially more duplicated orthologs compared with the three other diploid species.

Repeat-masking was performed on the culinary herb genome assemblies to mask repetitive elements (Supplementary Table S7). The proportion of masked bases was not dependent on genome assembly size, as *R. officinalis* (54.7%) and *O. basilicum* (61.6%) had a similar proportion of masked bases compared with *O. majorana* (65.5%) and *O. vulgare* (65.4%). Long terminal repeats (LTRs) were the most common repetitive elements, though the proportion of LTRs varied among the culinary herbs. LTRs represented 31.6% of the *R. officinalis* assembly compared with 47.8 and 49.3% of the *O. majorana* and *O. vulgare* assemblies. DNA elements were the second-most common classified element and represented 3.9% of the *O. vulgare* assembly up to 5.3% of the *R. officinalis* assembly. The proportion of long interspersed nuclear elements (LINEs) identified in the assemblies ranged from 0.28% (*O. majorana*) to 1.2% (*O. vulgare).* Instead, *O. majorana* had noticeably higher amounts of short interspersed nuclear elements (SINEs) identified (*n* = 3,469) compared with the other assemblies containing 209, 420, and 600 SINEs for *R. officinalis*, *O. basilicum*, and *O. vulgare*, respectively. The number of satellite repeats was associated with assembly size, though they did not represent >1.1% of the assembly size in any culinary herb.

### 3.2. Mono- and sesqui-terpene profiles of culinary herbs

**Figure 1.**
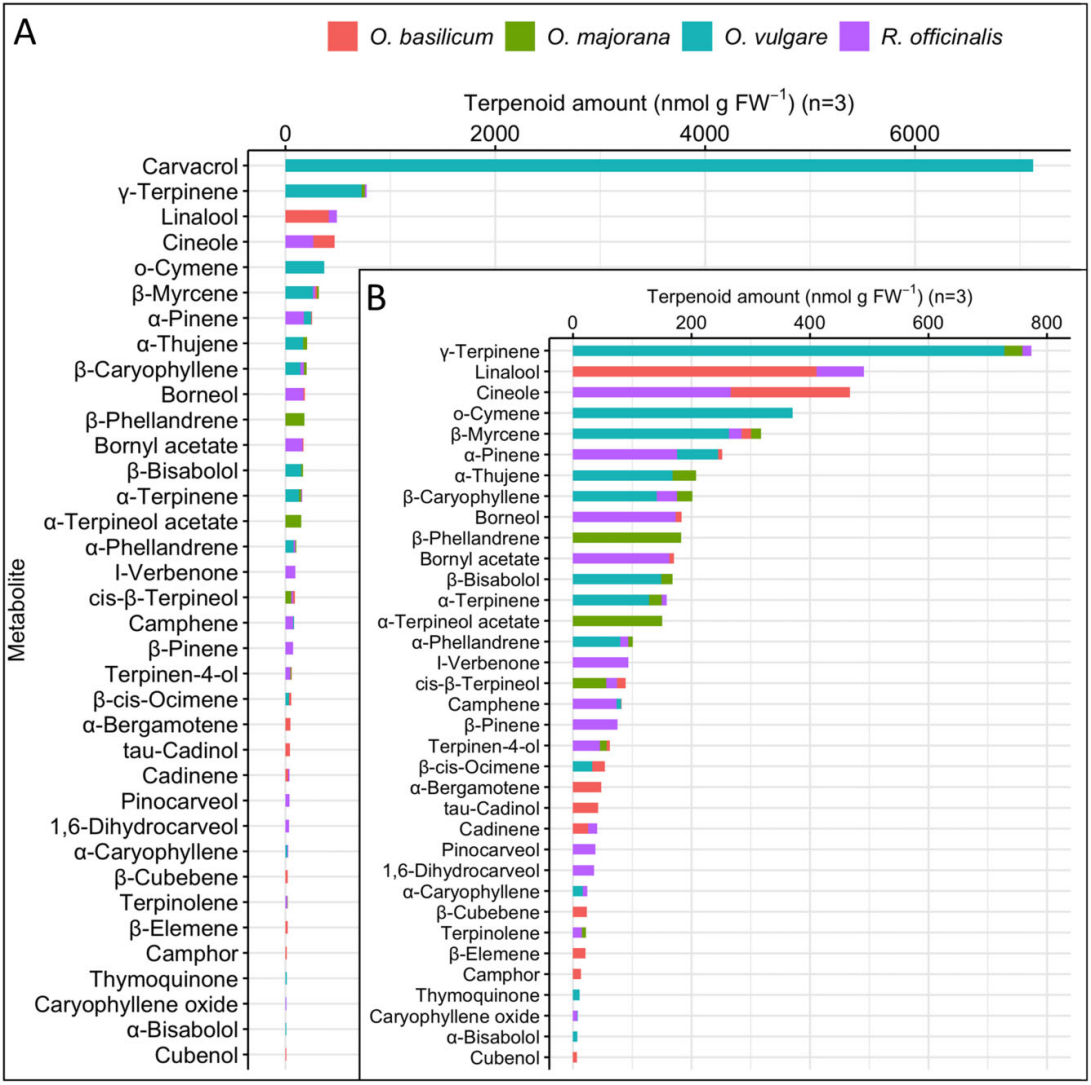
Terpenoid profiles in four culinary herbs. Leaf tissue from four culinary herbs was subjected to targeted metabolite profiling. Terpenoid levels are the average of three replicates, measured in nmol per gram of fresh weight. (A) Distribution of metabolites including carvacrol. (B) Distribution of metabolites excluding carvacrol.

As terpenoids are produced primarily in the leaves,^60^ metabolite profiling of leaf terpenoids from the four species was performed by GC−MS (Fig. 1). Spectrometric analyses revealed that these plants produce both monoterpenes and sesquiterpenes, with monoterpenes contributing to a higher degree (Supplementary Table S8). A total of 25 different monoterpenes were identified with only β-myrcene produced in all cultivars. Ten monoterpenes were species-specific (e.g. carvacrol was found only in *O. vulgare*), while the others, such as γ-terpinene, were shared by two or three species. The highest monoterpene diversity was detected in *R. officinalis*, which produced 17 monoterpenes, while the other cultivars synthesized 10 − 11 compounds. The total amounts of produced monoterpenes also varied between the species, ranging from 8.99 µmol g FW^−1^ in *O. vulgare* to just 0.52 µmol g FW^−1^ in *O. majorana* (Supplementary Table S8). The obtained metabolic profiles were generally consistent with literature reports.^61–64^

**Figure 2.**
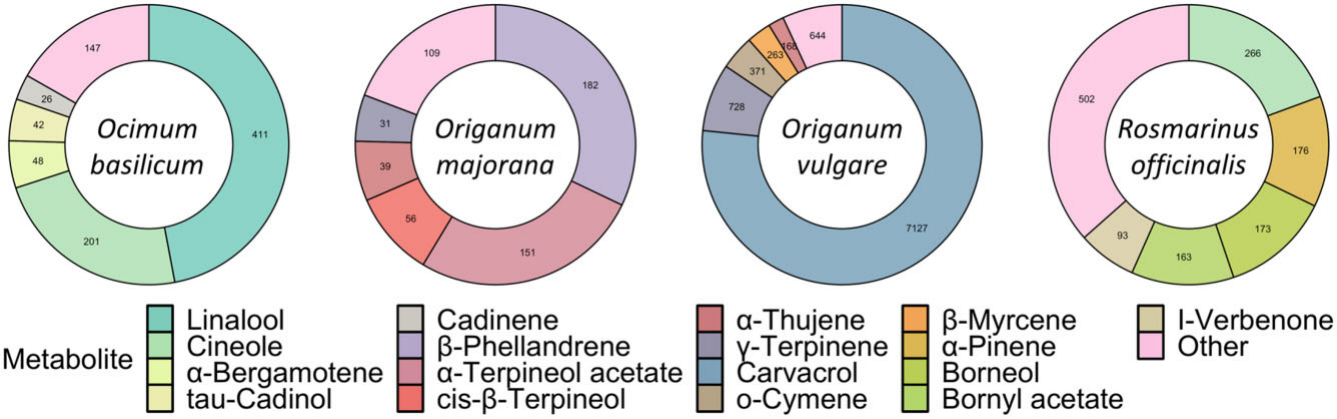
Unique terpenoid profiles in leaf tissue of four culinary herbs. The five most detected terpenoids are indicated for each culinary herb, and remaining terpenoids are classified as ‘Other’. Terpenoid levels are the average of three replicates, measured in nmol per gram of fresh weight.

In contrast to rich chemical diversity observed for monoterpenes, the amount and spectrum of sesquiterpenes was significantly lower. A total of eleven sesquiterpenes were detected, five of which were unique to a single species. There was no sesquiterpene shared by all four species, although β-caryophyllene was produced by three species. While *O. basilicum* produced the most diverse spectrum of sesquiterpenes, the highest amount of sesquiterpenes was found in *O. vulgare*, suggesting that this species is the highest producer of both mono- and sesquiterpenes. Comparative analysis of the most abundant compounds revealed that in species with relatively high levels of terpenoids, *O. vulgare* and *R. officinalis*, these are mostly monoterpenes, while in low terpene producers, such as *O. basilicum* and *O. majorana*, sesquiterpenes contribute to the overall terpenoid profile. This analysis also revealed that the spectra of most abundant compounds are mostly species-specific (Fig. 2).

### 3.3. Orthologous and paralogous clustering

**Figure 3.**
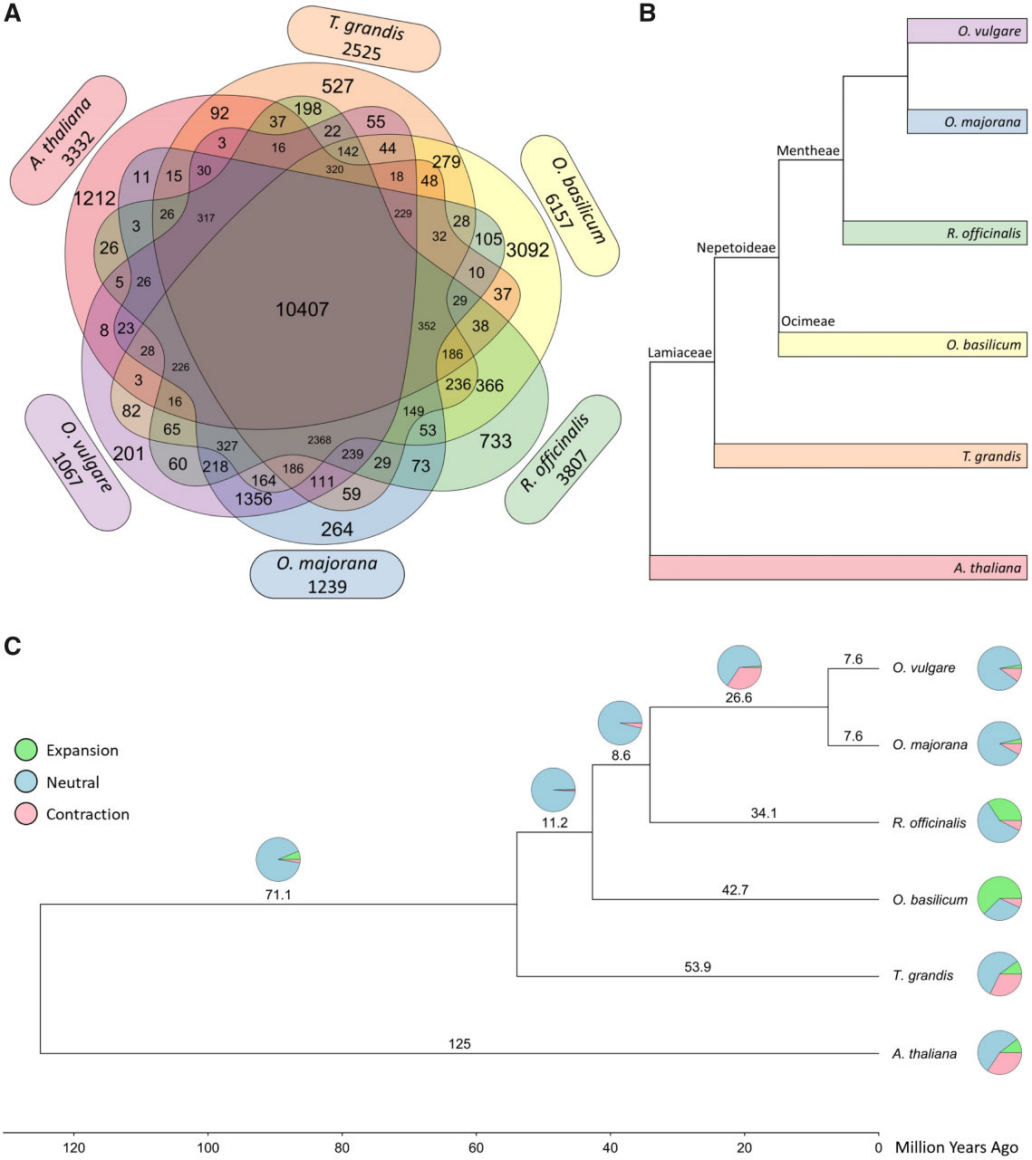
Orthologous relationships between four culinary herbs. (A) Venn diagram of orthologous groups shared among species. Numbers next to the species and outside of the plot indicate the number of singleton genes. (B) Cladogram of the species’ evolutionary relationships. Species belonging to the Lamiaceae family are further divided into the Nepetoideae subfamily and Mentheae and Ocimeae clades (Eltsholtzieae clade is not represented here). (C) Gene family evolution of four Nepetoideae culinary herbs and outgroups. Using a root age of 125 million years ago, the species were estimated to share 12,615 gene families. Changes to gene family sizes for each lineage are indicated by pie charts, where green indicates expansion, blue indicates neutrality, and red indicates contraction.

Orthofinder2 is a software programme that partitions genes according to their phylogenetic ancestry^46^ and clusters them into orthologous (orthogroups) and paralogous clusters. Identification and comparison of orthologs within orthogroups may reveal gene duplication or loss over evolutionary time. Thus, we performed this type of analysis for the four Nepetoideae species used in this study along with two additional species included in the analysis as outgroups. *Tectona grandis* (teak) was used as a non-Nepetoideae Lamiaceae species along with the model species *A. thaliana*, a member of the Brassicaceae. High confidence representative predicted peptides for these six species, along with curated Lamiaceae TPS obtained from SwissProt, were used as input for Orthofinder2; in total, 219,047 predicted peptides were included. Of these, 200,920 (91.7%) were assigned to 25,660 orthogroups (Fig. 3A;Supplementary Dataset S2).

Orthogroup occupancy by species was similar for the Lamiaceae species, ranging from 65.5% (*T. grandis*) to 76.6% (*O. basilicum*), while orthologous genes from the non-Lamiaceae outgroup *A. thaliana* were only present in 53.9% of orthogroups. A rooted species tree^47^^,^^48^ revealed a topology in agreement with a previous Lamiaceae cladogram (Fig. 3B).^3^ As expected, *O. majorana* and *O. vulgare* were closely related, and teak and *A. thaliana* were more distantly related to the rest of the Nepetoideae species.

In total, 10,407 orthologous groups contained orthologs from all 6 species (Supplementary Dataset S2). Genes in these groups were enriched in core biological processes and molecular function such as translation (GO : 0006412; *p* < 1e−30), intracellular protein transport (GO: 0006886; *p* < 1e−30), and structural constituent of ribosome (GO: 0003735; *p* < 1e−30). Lamiaceae members shared 2,368 orthologous groups containing genes involved in oxidation-reduction (GO: 0055114; *p* < 1e−30), protein phosphorylation (GO: 0006468; *p* < 1e−30), regulation of transcription (GO: 0006355; *p* < 1e−30), defence response (GO: 0006952; *p* < 1e−30), and TPS activity (GO: 0010333; *p* = 2.3e−13), among others. Of the orthologous genes unique to the culinary herbs and their singletons (Supplementary Table S9), the most-significant biological processes were recognition of pollen (GO: 0048544; *p* = 1.5e−28) and translation (GO: 0006412; *p* = 3.6e−06). These genes were associated with cellular locations such as the ribosome (GO: 0005840; *p* = 1.7e−07) and nucleosome (GO: 0000786; *p* = 0.00056). The most significant molecular functions for this subset of genes were protein serine/threonine kinase activity (GO: 0004674; *p* = 7.3e−16), ADP binding (GO: 0043531; *p* = 1.2e−14), and TPS activity (GO: 0010333; *p* = 5.80e−11). Considering that divalent metal cofactors have been shown to influence TPS activity and specificity,^65^^,^^66^ other notably enriched terms among the culinary herb-specific orthologous genes included magnesium ion binding (GO: 0000287; *p* = 5.6e−05), manganese ion binding (GO: 0030145; *p* = 0.00068), and copper ion binding (GO: 0005507; *p* = 0.00994).

### 3.4. Gene family analysis

Of the 25,660 orthologous groups identified by the Orthofinder analysis, 12,615 were inferred to be present in the most recent common ancestor and were used in the CAFE analysis along with the ultrametric tree. The number of gene families in the observed Lamiaceae species was found to be generally consistent over evolutionary time (Fig. 3C). Compared with the rest of the culinary herbs, a noticeable gene family contraction occurred in the *Origanum* genus, while *O. basilicum* shows significant gene family expansion, likely due in part to its tetraploidy. Both non-Nepetoideae outgroups share a similar number and proportion of contracted gene families.

To better understand evolutionary relationships of these four culinary herbs among the ever-growing list of sequenced Lamiaceae spp., we conducted an Orthofinder analysis for all available Lamiaceae predicted proteomes (Supplementary Dataset S1 and Fig. S2), characterizing 34,998 orthologous groups. Approximately 30% of the orthologous groups contained at least one ortholog from each species. Intra-genus orthologous groups for the *Origanum* spp. and *Nepeta* spp. contained the second- and third-most number of non-encompassing intersections. Orthologs from *Pogostemon cablin*, an octoploid, were represented in the most orthologous groups. The species tree derived from ancestral gene families was in agreement with previous cladograms,^3^^,^^4^^,^^67^ confirming the monophyly of Nepetoideae subfamily and the polyphyly of the *Salvia* genus, as described previously.^68^

### 3.5. Identification of precursor genes and TPS in four culinary herbs

**Figure 4.**
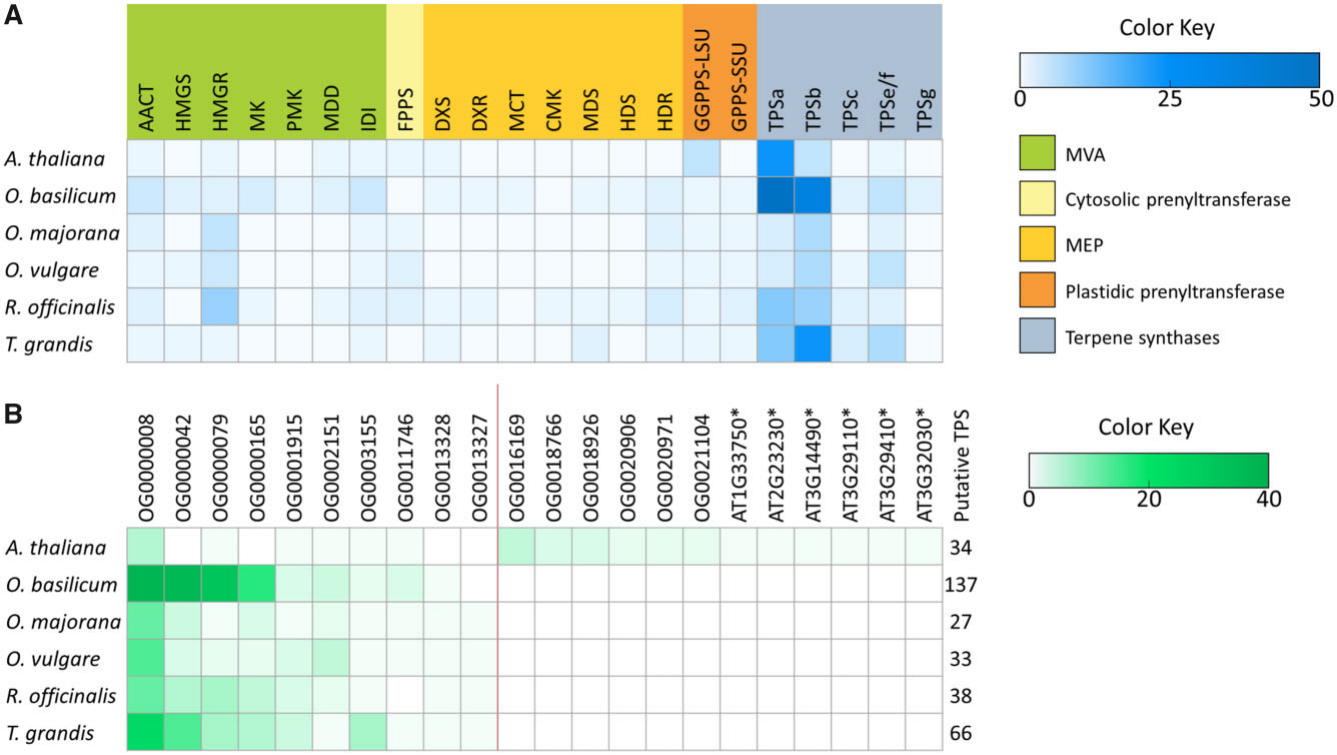
Orthologous gene clusters in the terpenoid biosynthetic pathway. The number of corresponding orthologs from each species is indicated by colour gradient intensity. (A) Orthogroups occupied by *Arabidopsis thaliana* genes involved in the mevalonic acid (MVA) and methylerythriol phosphate (MEP) pathways. MVA pathway leading to sterols and sesquiterpenes: acetyl-CoA acetyltransferase activity (AACT); 3-hydroxy-3-methylglutaryl coenzyme A synthase (HMGS); hydroxymethylglutaryl-CoA reductase (HMGR); ATP: mevalonate phosphotransferase (MK); phosphomevalonate kinase (PMK); mevalonate diphosphate decarboxylase (MDD); isopentenyl diphosphate delta-isomerase (IDI); geranyltranstransferase (FPPS). MEP pathway leading to geraniol, monoterpenes, and diterpenes: 1-deoxy-D-xylulose-5-phosphate synthase (DXS); 1-deoxy-d-xylulose-5-phosphate reductoisomerase (DXR); 2-C-methyl-d-erythritol 4-phosphate cytidylyltransferase (MCT); 4-(cytidine 50-diphospho)-2-C-methyl-d-erythritol kinase (CMK); 2-C-methyl-d-erythritol 2,4-cyclodiphosphate synthase (MDS); 4-hydroxy-3-methylbut-2-en-1-yl diphosphate synthase (HDS); 1-hydroxy-2-methyl-2-(E)-butenyl 4-diphosphate reductase (HDR); geranylgeranyl pyrophosphate synthase large subunit (GGPPS-LSU); geranyl pyrophosphate synthase small subunit (GPPS-SSU). Terpene synthase subfamilies: TPS a, b, c, g, e/f. (B) Orthogroups occupied by *A. thaliana* and Lamiaceae terpene synthase genes. Orthologous groups specific to *A. thaliana* are shown to the right of the red divider line; singletons are denoted by an ‘*’ following the gene ID.

Terpenes are synthesized from common IPP and DMAPP precursors via the MEP and MVA pathways. To examine the terpenoid biosynthetic pathway in the culinary herbs, orthologous groups were queried for genes belonging to *A. thaliana* MEP and MVA pathways, as identified previously (Supplementary Table S10).^3^ All 22 of these *A. thaliana* MEP/MVA genes clustered into 17 orthologous groups (Supplementary Table S11). Six additional *A. thaliana* genes also clustered with the MEP/MVA orthogroups OG0001733 and OG0006021, representing five geranylgeranyl phosphate synthases and a putative 1-deoxyxylulose 5-phosphate synthase, respectively. A total of 148 culinary herb orthologs were present among the MEP/MVA orthologous groups. In 13 of the 17 orthogroups, each culinary herb contained equal to or greater numbers of orthologs than *A. thaliana*. However, the difference in the number of MEP/MVA orthologs across all species was small as each culinary herb contained one to ten MEP/MVA orthologs, compared with one to six orthologs in *A. thaliana* and one to three orthologs in *T. grandis* (Fig. 4A).

TPS enzymes synthesize terpenoids from the GPP and FPP end products of the MEP and MVA pathways. To identify TPS genes in the four culinary herbs, orthogroup occupancy of previously published *A. thaliana* TPSs^3^ as well as curated Lamiaceae TPSs was investigated. The *A. thaliana* TPSs (*n* = 34) belong to TPS subfamilies TPSa, TPSb, TPSc, TPSe/f, and TPSg; these genes clustered into 12 orthologous groups and 6 singletons (Table 3; Supplementary Table S12). Six of the 12 orthologous groups containing *A. thaliana* TPSs were unique to *A. thaliana*; the other 6 orthologous groups contained a total of 151 putative TPSs from the four culinary herbs. Curated Lamiaceae TPSs (*n* = 26) were also included in the analyses to identify Lamiaceae-specific TPSs (Supplementary Table S1); these ‘bait’ Lamiaceae TPS genes clustered into 7 orthologous groups containing a total of 212 putative TPS across the culinary herbs (Table 3). Within these orthogroups, *O. basilicum* contained the most TPSs (*n* = 128) of the culinary herbs, followed by *R. officinalis* (*n* = 35), *O. vulgare* (*n* = 26), and *O. majorana* (*n* = 23). In these same orthogroups, 57 orthologs from *T. grandis* were detected along with 8 orthologs from *A. thaliana*. Orthologous groups OG0000008, OG0000079, and OG0001915 contained TPSs from both *A. thaliana* and Lamiaceae bait TPSs, representing TPS subfamilies TPSa, TPSb, and TPSc, respectively.

**Table 3:** Orthogroup occupancy of *Arabidopsis thaliana* and Lamiaceae terpene synthase genes

Orthogroup	*Arabidopsis thaliana*	TPS bait	*Ocimum basilicum*	*Origanum majorana*	*Rosmarinus officinalis*	*Tectona grandis*	*Origanum vulgare*	Total	Subfamily
OG0000008	6	12	38	12	12	24	14	118	TPSb
OG0000079	1	1	32	1	7	7	2	51	TPSa
OG0001915	1	1	3	1	3	4	3	16	TPSc
OG0000042	0	3	37	4	6	14	3	67	−
OG0000165	0	4	17	3	5	6	2	37	−
OG0013327	0	3	0	1	1	1	1	7	−
OG0013328	0	2	1	1	1	1	1	7	−
OG0002151	1	0	4	2	2	1	5	15	TPSe/f
OG0003155	1	0	2	1	1	7	1	13	TPSe/f
OG0011746	1	0	3	1	0	1	1	7	TPSg
OG0016169	5	0	0	0	0	0	0	5	TPSa
OG0018766	3	0	0	0	0	0	0	3	TPSa
OG0018926	3	0	0	0	0	0	0	3	TPSa
OG0020906	2	0	0	0	0	0	0	2	TPSa
OG0020971	2	0	0	0	0	0	0	2	TPSa
OG0021104	2	0	0	0	0	0	0	2	TPSa
OG0026006	1	0	0	0	0	0	0	1	TPSa
OG0026746	1	0	0	0	0	0	0	1	TPSa
OG0027218	1	0	0	0	0	0	0	1	TPSa
OG0027424	1	0	0	0	0	0	0	1	TPSa
OG0027428	1	0	0	0	0	0	0	1	TPSa
OG0027452	1	0	0	0	0	0	0	1	TPSa

Genes below the line were unique to *A. thaliana*.

Overall, the culinary herb genomes encoded a total of 235 putative TPS occupying ten orthologous groups with TPS from *A. thaliana* or the Lamiaceae bait. Nearly four to five times as many TPSs were found in *O. basilicum* (*n* = 137) compared with the diploid culinary herbs that contained substantially fewer TPSs: *R. officinalis* (*n* = 38), *O. vulgare* (*n* = 33), *O. majorana* (*n* = 27) (Fig. 4B). Consistent with the relatively high levels and rich chemical diversity of monoterpenes in these culinary herbs (Supplementary Table S8), these TPSs were mainly represented by members of the TPSb subfamily, which includes most of the angiosperm monoterpene synthases.^69^

Among the six orthogroups jointly occupied by *A. thaliana* TPSs and culinary herb orthologs, five orthogroups contained Lamiaceae orthologs in the same or greater quantity than the *A. thaliana* TPSs (Table 3). Whereas *A. thaliana* contained one to six orthologs in both the MVA/MEP and TPS related orthogroups, the culinary herbs generally contained more TPS orthologs compared with MEP/MVA orthologs (Fig. 4; Supplementary Table S11). For example, orthogroup OG0000008 contained six *A. thaliana* TPSs, all belonging to the TPSb subfamily, while all other mint species were represented by 12 (*O. majorana* and *R. officinalis*) to 38 (*O. basilicum*) orthologs. In OG0000079, a single *A. thaliana* sesquiterpene synthase gene, TPS21 (AT5G23960) was present along with one and two orthologs in *O. majorana* and *O. vulgare*, respectively, and 32 orthologs in *O. basilicum*. However, this trend did not hold for all orthologous groups. For example, the highest number of orthologs in orthogroups OG0003155 (*n* = 7) and OG0001915 (*n* = 4) belonged to *T. grandis*; the *A. thaliana* TPSs in these orthogroups were associated with TPSe/f and TPSc subfamilies, respectively. Lineage-specific *A. thaliana* TPS orthogroups included OG0016169 (*n* = 5), OG0018766 (*n* = 3), OG0018926 (*n* = 3), OG0020906 (*n* = 2), OG0020971 (*n* = 2), and OG0021104 (*n* = 2) in addition to the six singletons.

### 3.6. Physical clustering of specialized metabolite pathways

Physical clustering within the genome has been reported for numerous specialized metabolism biosynthetic pathways,^70^ thus to identify putative clusters of enzymes involved in secondary metabolite synthesis, plantiSMASH v1.0 analysis^71^ was performed on each culinary herb genome assembly with its high confidence representative gene set (Supplementary Table S13). The quantity and classification of clusters detected varied by species. In total, there were 104 clusters detected in *O. basilicum*, 36 clusters detected in *O. majorana*, 38 clusters detected in *O. vulgare*, and 22 clusters detected in *R. officinalis*. In particular, the four species were enriched in clusters related to terpene and saccharide production. Among the culinary herbs, the most terpene clusters were found for the *O. basilicum* assembly, with 226 genes located across 24 clusters. The *O. majorana* and *O. vulgare* assemblies contained eight and nine terpene clusters, with 65 and 83 corresponding genes, respectively. The smallest number of terpene clusters was found in *R. officinalis*, with 26 genes in 2 clusters. In comparison, *A. thaliana* had 7 putative terpene clusters containing 129 genes, and *T. grandis* had 6 clusters with 72 genes. Other secondary metabolite clusters were represented by a combination of terpene-related genes along with other secondary metabolites such as alkaloids, lignans, and polyketides.

## 4. Conclusion 

Plants in the mint family are used worldwide for their unique chemical profiles conferred by specialized metabolites such as terpenoids. In this study, we focussed on four mint species commonly utilized as culinary herbs: *O. basilicum*, *O. majorana*, *O. vulgare*, and *R. officinalis*. Genome sequencing, assembly, and annotation of these herbs revealed a diversity of genes involved in terpenoid biosynthesis. In addition, targeted metabolic profiling revealed the diversity of monoterpenes and sesquiterpenes in these species and exemplified unique terpenoid profiles for each species. Our study showcases the genomic and metabolomic characterization of these four herbs that can be used to further explore terpene biosynthesis.

## Supplementary data

Supplementary data are available at *DNARES* online.

## Supplementary Material

dsaa016_Supplementary_Data
