# Corrigendum to: Genome sequencing of four culinary herbs reveals terpenoid genes underlying chemodiversity in the Nepetoideae

**DOI:** 10.1093/dnares/dsaa025

**Published:** 2020-11-13

**Authors:** Nolan Bornowski, John P Hamilton, Pan Liao, Joshua C Wood, Natalia Dudareva, C Robin Buell

**Affiliations:** Department of Plant Biology, Michigan State University, East Lansing, MI 48824, USA; Department of Plant Biology, Michigan State University, East Lansing, MI 48824, USA; Department of Biochemistry, Purdue University, West Lafayette, IN 47907-2063, USA; Department of Plant Biology, Michigan State University, East Lansing, MI 48824, USA; Department of Biochemistry, Purdue University, West Lafayette, IN 47907-2063, USA; Department of Plant Biology, Michigan State University, East Lansing, MI 48824, USA; Plant Resilience Institute, Michigan State University, East Lansing, MI 48824, USA; MSU AgBioResearch, Michigan State University, East Lansing, MI 48824, USA

*DNA Research*, 2020. doi: 10.1093/dnares/dsaa016

In the originally published version of this manuscript, Figure 3C. depicts an ultrametric tree showing gene family evolution and estimated species divergence times. In the published version of this figure, some branch labels (divergence times) and pie charts were inadvertently switched. The authors calculated the divergence times and pie charts correctly. However, an error was made in the plotting of the ultrametric tree when the branches were rotated to be concordant with the cladogram in panel B. This single mistyped variable in the R code resulted in the branches being rotated but the labels and pie charts were not. The correct branch labels in order of divergence are: *O. basilicum*: 42.7; ancestral branch of *R. officinalis & Origanum* spp.: 8.6; *R. officinalis*: 34.1; ancestral branch of *Origanum* spp.: 26.6; *O. vulgare*: 7.6, *O. majorana*: 7.6.

The authors apologize for this error which, has now been corrected online.

