# Supplementary material for: Genome sequencing of four culinary herbs reveals terpenoid genes underlying chemodiversity in the Nepetoideae
Source: DNA Res. 2020 Jul 31;27(3):dsaa016. doi: 10.1093/dnares/dsaa016 (PMC7508350; doi:10.1093/dnares/dsaa016)
Supplement: dsaa016_Supplementary_Data [file dsaa016_supplementary_data.zip › Supplementary Information.pdf]

Supplementary Information for

Genome sequencing of four culinary herbs reveals terpenoid genes underlying chemodiversity in the Nepetoideae

Nolan Bornowski,[1] John P. Hamilton,[1] Pan Liao,[2] Joshua C. Wood,[1] Natalia Dudareva,[2] and C. Robin Buell[1,3,4]*

[1]Department of Plant Biology, Michigan State University, East Lansing, MI 48824, USA

[2]Department of Biochemistry, Purdue University, West Lafayette, IN 47907-2063, USA

[3]Plant Resilience Institute, Michigan State University, East Lansing, MI 48824, USA

[4]MSU AgBioResearch, Michigan State University, East Lansing, MI 48824, USA

*To whom correspondence should be addressed: C. Robin Buell (buell@msu.edu)

This PDF file includes:

Supplementary Figure Legends

Supplementary Figures

Supplementary Tables Legends

Supplementary Dataset Legends

References

Other supplementary materials for this manuscript include the following:

Supplementary Tables S1 to S13

Supplementary Datasets 1 and 2

Supplementary Figure Legends

Supplementary Figure 1. Jellyfish k-mer analysis.

K-mer estimated genome sizes were determined for all four species using Jellyfish v2.2.0 (Marçais and Kingsford 2011) with a k-mer size of 31 and adjusted for heterozygous sequences (light blue dashed line) using the R package findGSE v0.1.0 (Sun et al. 2017). The grey line indicates the observed number of k-mers, the light blue line indicates the distribution of heterozygous sequence, the dark blue line indicates the model-fitted k-mer distribution, and the red line indicates the final fitting.

A: *O. basilicum;* B: *O. majorana;* C: *O. vulgare;* D: *R. officinalis*

Supplementary Figure 2. Orthologous gene relationships among Lamiaceae species.

Predicted proteomes for all available Lamiaceae species (Supplementary Dataset 1) were assigned orthologous groups using Orthofinder v2.3.7 (Emms and Kelly 2018a). Out of 34,998 orthogroups, 30% of orthogroups contained an ortholog from all species. The four culinary herbs presented in this study are indicated by blue text. The bar plot shows the number of orthogroups containing orthologs from the species intersections indicated by the dot plot below. The cladogram was constructed and rooted from ancestral orthologous groups with the STAG (Emms and Kelly 2018b) and STRIDE (Emms and Kelly 2017) algorithms, respectively.

Supplementary Figures

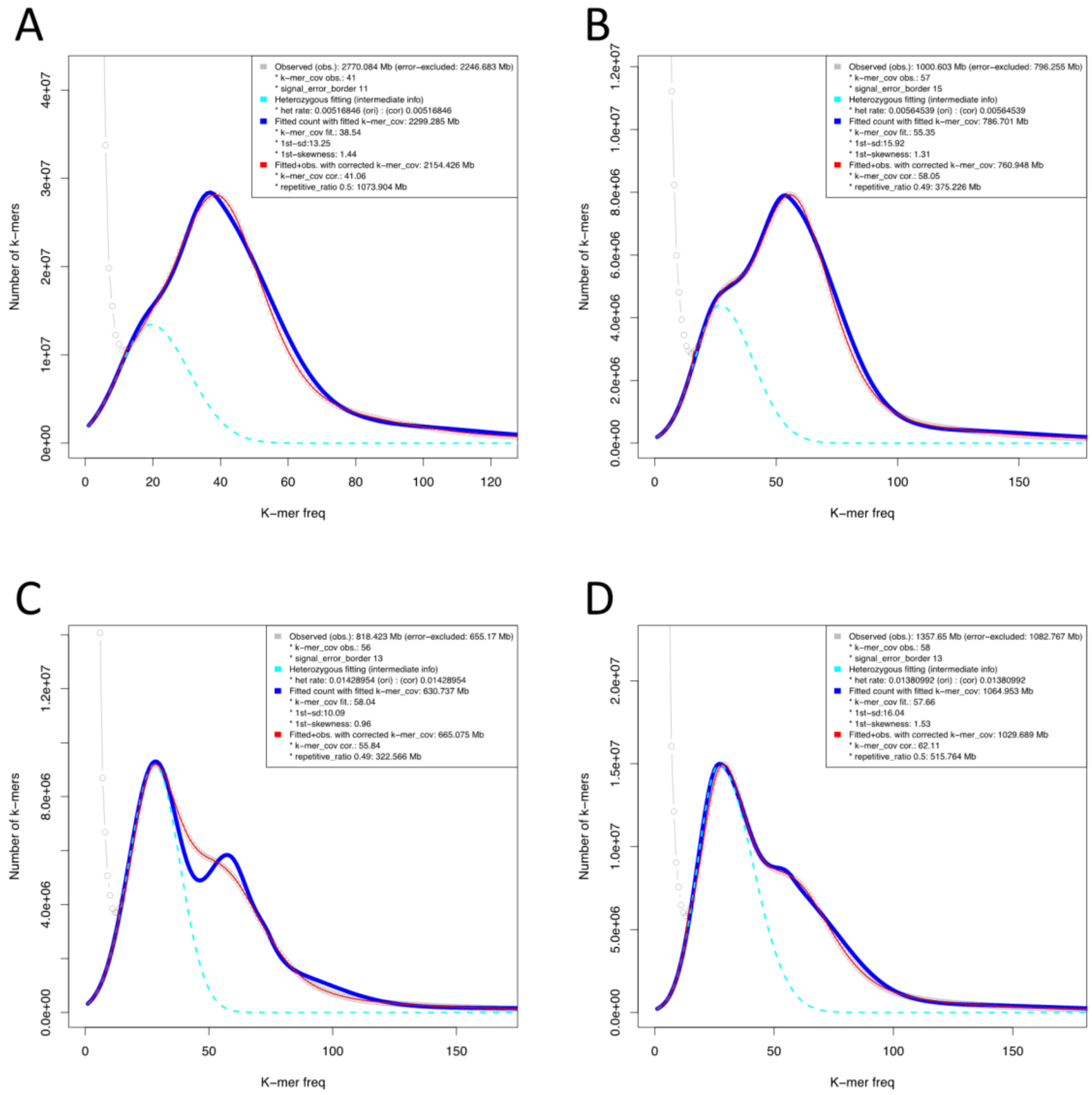

Supplementary Figure 1. Jellyfish k-mer analysis.

K-mer estimated genome sizes were determined for all four species using Jellyfish v2.2.0 (Marçais and Kingsford 2011) with a k-mer size of 31 and adjusted for heterozygous sequences (light blue dashed line) using the R package findGSE v0.1.0 (Sun et al. 2017). The grey line indicates the observed number of k-mers, the light blue line indicates the distribution of heterozygous sequence, the dark blue line indicates the model-fitted k-mer distribution, and the red line indicates the final fitting.

A: *O. basilicum;* B: *O. majorana;* C: *O. vulgare;* D: *R. officinalis*

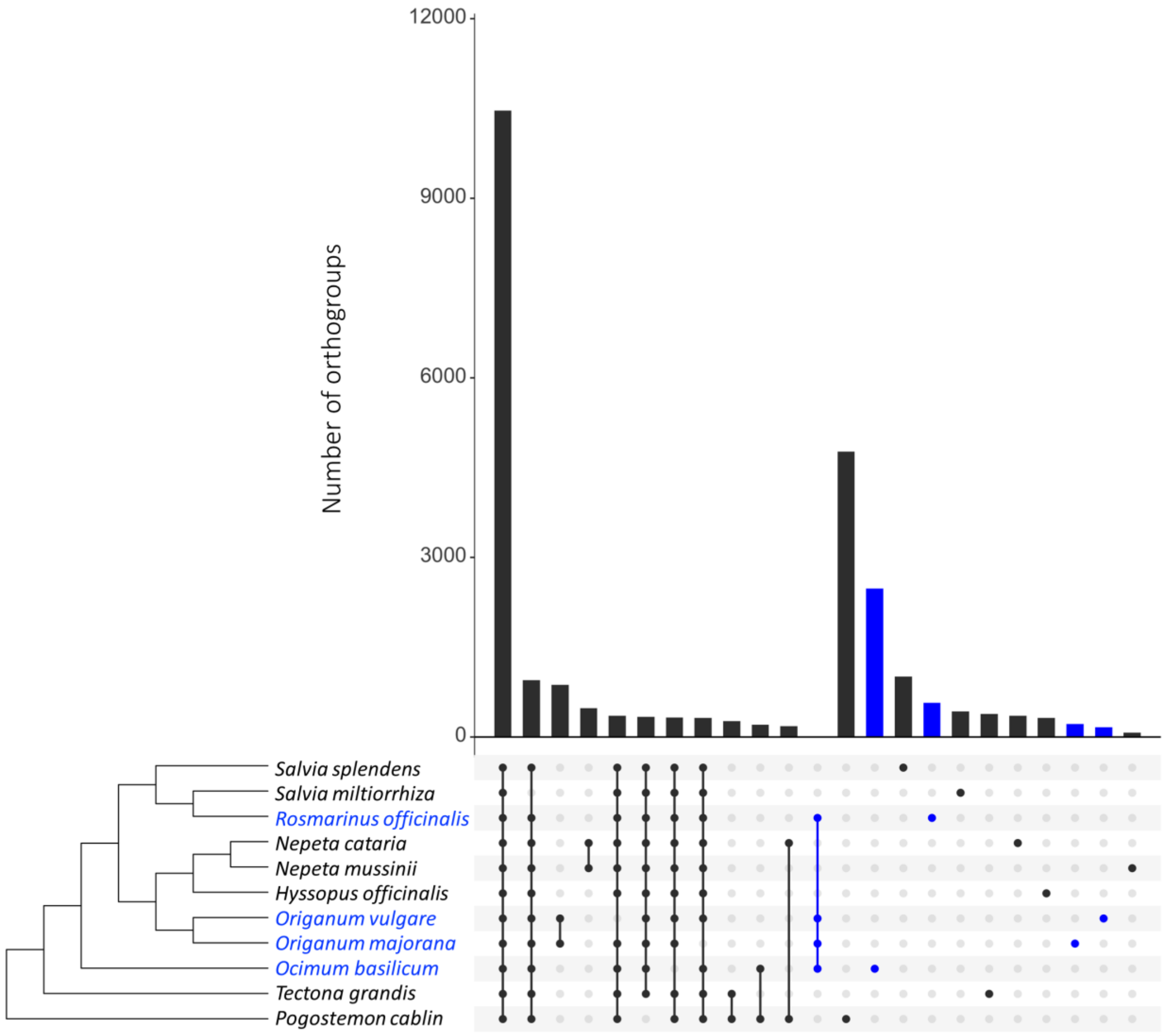

Supplementary Figure 2. Orthologous gene relationships among Lamiaceae species.

Predicted proteomes for all available Lamiaceae species (Supplementary Dataset 1) were assigned orthologous groups using Orthofinder v2.3.7 (Emms and Kelly 2018a). Out of 34,998 orthogroups, 30% of orthogroups contained an ortholog from all species. The four culinary herbs presented in this study are indicated by blue text. The bar plot shows the number of orthogroups containing orthologs from the species intersections indicated by the dot plot below. The cladogram was constructed and rooted from ancestral orthologous groups with the STAG (Emms and Kelly 2018b) and STRIDE (Emms and Kelly 2017) algorithms, respectively.

Supplementary Table Legends

Supplementary Table S1. Curated TPS transcripts from Lamiaceae species used as bait to identify orthologous genes

Supplementary Table S2. Genome assembly size and estimation for four culinary herbs

Supplementary Table S3. 10x Genomics libraries used in this study

Supplementary Table S4. RNA-sequencing libraries used in this study

Supplementary Table S5. Comparison of *O. basilicum* assemblies

Supplementary Table S6. BUSCO assessments of gene annotation for four culinary herb genomes

Supplementary Table S7. Repeat composition of four culinary herbs

Supplementary Table S8. GC-MS data for terpenoids in leaves of four culinary herbs

Sup. Table 9. GO term enrichment results for genes in culinary herb-specific orthologous groups and their singletons compared to all genes from the 6 species

Supplementary Table S10. Methylerythriol phosphate (MEP) and mevalonic acid (MVA) pathway genes from *Arabidopsis thaliana*

Supplementary Table S11. Orthogroup occupancy of *Arabidopsis thaliana* methylerythriol phosphate (MEP) and mevalonic acid (MVA) genes

Supplementary Table S12. Orthogroup occupancy of *Arabidopsis thaliana* terpene synthases

Supplementary Table S13. Specialized metabolite gene clusters identified by plantiSMASH

Supplementary Dataset Legends

Supplementary Dataset 1. Published Lamiaceae genomes.

This file contains a table of publications describing the sequencing and assembly of available Lamiaceae genomes. Scaffold assembly sizes and N50 sizes are provided for each entry. References are provided for assembly and annotation datasets as available.

Supplementary Dataset 2. Orthologous groups for four culinary herbs, *Tectona grandis*, and *Arabidopsis thaliana*.

T. grandis served as a Nepetoideae outgroup, and *A. thaliana* served as a Lamiaceae outgroup. Orthofinder2 v2.3.7 (Emms and Kelly 2018a) was run with default parameters using representative peptides for all six species to generate 25,660 orthologous groups (not including singletons).

References

Emms DM, Kelly S (2018a) OrthoFinder2: fast and accurate phylogenomic orthology analysis from gene sequences. bioRxiv. doi: 10.1101/466201

Emms DM, Kelly S (2018b) STAG: Species Tree Inference from All Genes. bioRxiv 267914. doi: 10.1101/267914

Emms DM, Kelly S (2017) STRIDE: Species Tree Root Inference from Gene Duplication Events. Mol Biol Evol 34:3267–3278. doi: 10.1093/molbev/msx259

Marçais G, Kingsford C (2011) A fast, lock-free approach for efficient parallel counting of occurrences of k-mers. Bioinformatics 27:764–770. doi: 10.1093/bioinformatics/btr011

Sun H, Ding J, Piednoël M, Schneeberger K (2017) findGSE: estimating genome size variation within human and Arabidopsis using k-mer frequencies. Bioinformatics 34:550–557. doi: 10.1093/bioinformatics/btx637
