# Supplementary figures and images for: Genome sequencing of four culinary herbs reveals terpenoid genes underlying chemodiversity in the Nepetoideae
Source: DNA Res. 2020 Jul 31;27(3):dsaa016. doi: 10.1093/dnares/dsaa016 (PMC7508350; doi:10.1093/dnares/dsaa016)

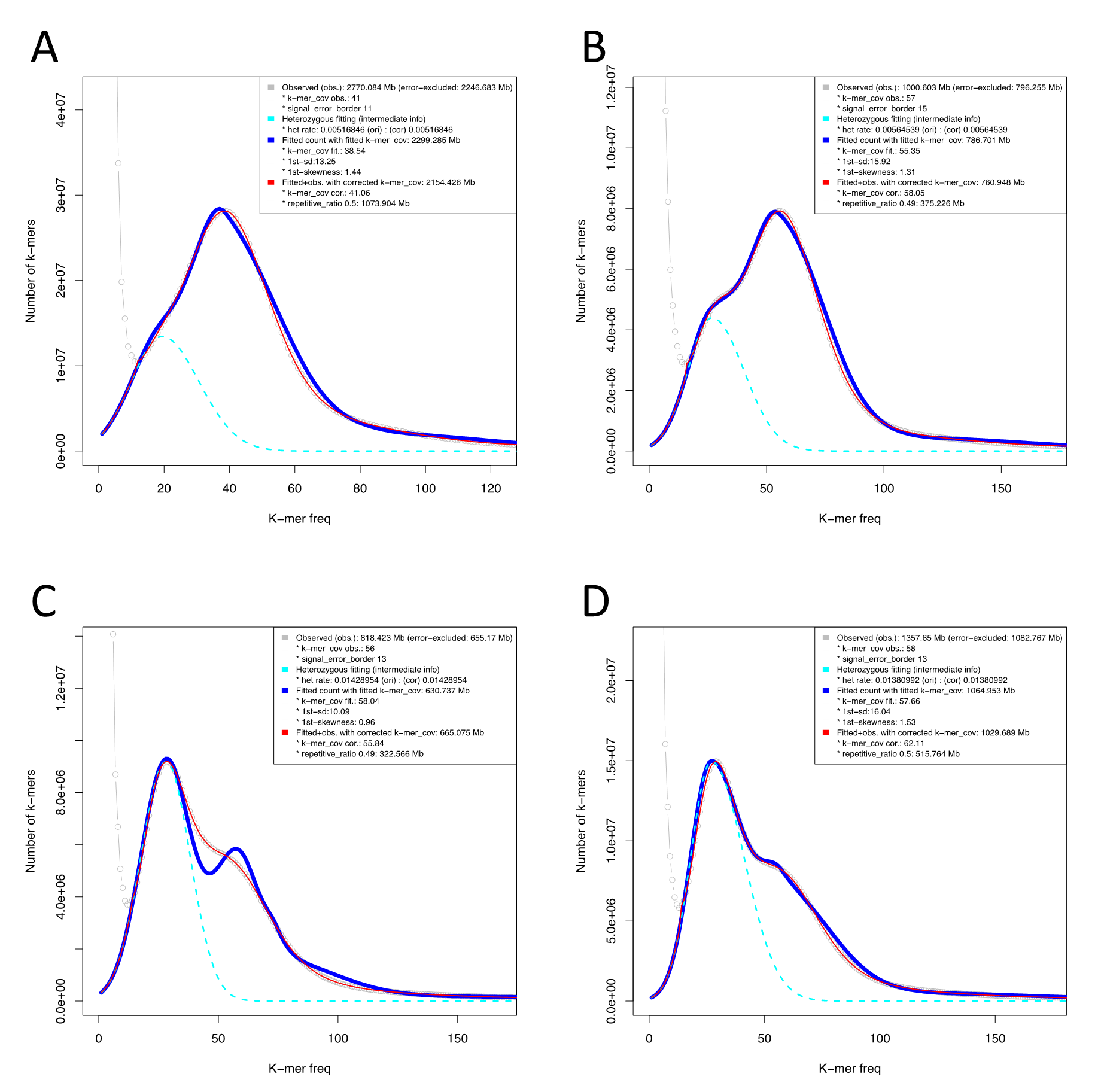

Supplement: dsaa016_Supplementary_Data [file dsaa016_supplementary_data.zip › Supplementary_Figure_1.tiff]

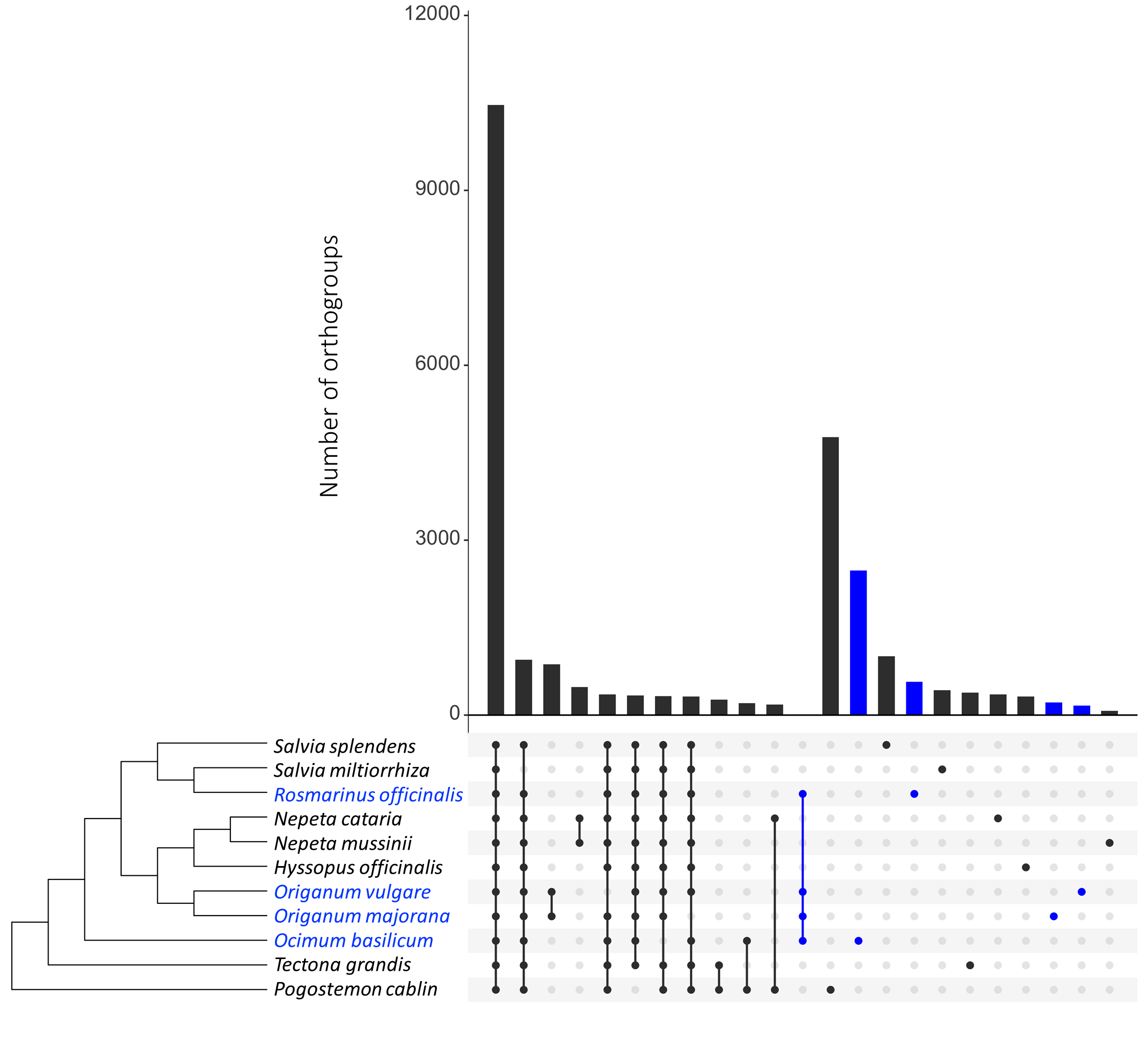

Supplement: dsaa016_Supplementary_Data [file dsaa016_supplementary_data.zip › Supplementary_Figure_2.tiff]
